# High AUF1 level in stromal fibroblasts promotes carcinogenesis and chemoresistance and predicts unfavorable prognosis among locally advanced breast cancer patients

**DOI:** 10.1186/s13058-022-01543-x

**Published:** 2022-07-11

**Authors:** Taher Al-Tweigeri, Noura N. AlRaouji, Asma Tulbah, Maria Arafah, Mouad Aboussekhra, Falah Al-Mohanna, Ahmed Mostafa Gad, Abdelmonneim M. Eldali, Tusneem A. Elhassan, Abdelilah Aboussekhra

**Affiliations:** 1 Oncology Center, King Faisal Specialist Hospital and Research Center, Riyadh, 11211 Saudi Arabia; 2 Department of Molecular Oncology, Cancer Biology and Experimental Therapeutics Section, King Faisal Specialist Hospital and Research Centre, MBC # 03, PO BOX 3354, Riyadh, 11211 Saudi Arabia; 3 Department of Pathology, King Faisal Specialist Hospital and Research Center, Riyadh, 11211 Saudi Arabia; 4 Department of Pathology, King Saud University, PO BOX 2925, Riyadh, 11461 Saudi Arabia; 5 Department of Comparative Medicine, King Faisal Specialist Hospital and Research Center, Riyadh, 11211 Saudi Arabia; 6 Clinical Oncology and Nuclear Medicine Department, Faculty of Medicine, Ain Shams University, Cairo, 11591 Egypt; 7 Department of Biostatistics, Epidemiology and Scientific Computing, King Faisal Specialist Hospital and Research Center, Riyadh, 11211 Saudi Arabia

**Keywords:** AUF1, Breast cancer, Cancer-associated fibroblasts, Prognosis

## Abstract

**Background:**

Locally advanced breast cancer (LABC), the most aggressive form of the disease, is a serious threat for women's health worldwide. The AU-rich RNA-binding factor 1 (AUF1) promotes the formation of chemo-resistant breast cancer stem cells. Thereby, we investigated the power of AUF1 expression, in both cancer cells and their stromal fibroblasts, as predictive biomarker for LABC patients’ clinical outcome following neoadjuvant treatment.

**Methods:**

We have used immunohistochemistry to assess the level of AUF1 on formalin-fixed paraffin-embedded tissues. Immunoblotting was utilized to show the effect of AUF1 ectopic expression in breast stromal fibroblasts on the expression of various genes both in vitro and in orthotopic tumor xenografts. Cytotoxicity was evaluated using the WST1 assay, while a label-free real-time setting using the xCELLigence RTCA technology was utilized to assess the proliferative, migratory and invasive abilities of cells.

**Results:**

We have shown that high AUF1 immunostaining (≥ 10%) in both cancer cells and their adjacent cancer-associated fibroblasts (CAFs) was significantly associated with higher tumor grade. Kaplan**–**Meier univariate analysis revealed a strong correlation between high AUF1 level in CAFs and poor patient’s survival. This correlation was highly significant in patients with triple negative breast cancer, who showed poor disease-free survival (DFS) and overall survival (OS). High expression of AUF1 in CAFs was also associated with poor OS of ER+/Her2− patients. Similarly, AUF1-positive malignant cells tended to be associated with shorter DFS and OS of ER+/Her2+ patients. Interestingly, neoadjuvant therapy downregulated AUF1 to a level lower than 10% in malignant cells in a significant number of patients, which improved both DFS and OS. In addition, ectopic expression of AUF1 in breast fibroblasts activated these cells and enhanced their capacity to promote, in an IL-6-dependent manner, the epithelial-to-mesenchymal transition and stemness processes. Furthermore, these AUF1-expressing cells enhanced the chemoresistance of breast cancer cells and their growth in orthotopic tumor xenografts.

**Conclusions:**

The present findings show that the CAF-activating factor AUF1 has prognostic/predictive value for breast cancer patients and could represent a great therapeutic target in order to improve the precision of cancer treatment.

**Supplementary Information:**

The online version contains supplementary material available at 10.1186/s13058-022-01543-x.

## Background

Breast cancer (BC) is the most common cancer among women and is the leading cause of cancer-related deaths worldwide [1]. BC is typically an extremely heterogeneous disease with high inter- and intra-tumor variabilities, which complicates diagnostics/prognostics as well as personalized therapy [2–4]. Locally advanced breast cancer (LABC) continues to be a serious health problem with adverse outcome despite all the revolutionary advancements made in cancer therapy and the introduction of precision medicine [5]. However, the prognostic of this aggressive form of BC has been improved through the introduction of some biological characteristics, which allow to choose the most suitable systemic treatments [5].

In addition to the heterogeneous composition of breast cancer, tumor cells are part of a very complex and dynamic ecosystem composed of various types of cells, hormones, cytokines, extracellular matrix and other factors [6]. Several lines of evidence indicate the presence of various cooperative signaling loops between cancer cells and their adjacent cancer-associated fibroblasts (CAFs), which influence the evolution and therapeutic response of cancer [7–9]. Thereby, it has become clear that CAFs and their biomarkers could be of great predictive/prognostic value. In this direction, we have recently shown that the RNA-binding protein AUF1 (also called heterogeneous nuclear ribonucleoprotein D, hnRNPD) plays a major role in the activation and the pro-carcinogenic effects of breast stromal fibroblasts [10]. In addition, AUF1 can promote the epithelial-to-mesenchymal transition (EMT) process as well as stemness in mammary epithelial cells [11]. Therefore, we sought to investigate here the potential use of the AUF1 expression level in breast cancer cells and their adjacent CAFs as prognostic tool for patients suffering LABC. The present findings present clear evidence that AUF1 expression level in breast CAFs has prognostic values for breast cancer patients, especially those diagnosed as TNBC. Furthermore, neoadjuvant therapy-dependent downregulation of AUF1 to a low level in tumor cells was shown to be associated with better survival. Additionally, we have shown that BSFs that express high level of AUF1 promote carcinogenesis and chemoresistance in a paracrine manner.

## Methods

### Cells and cell culture

Breast fibroblast cells were obtained and used as previously described [12]. MDA-MB-231 and MCF-7 cells were purchased in 2011 from ATCC and were authenticated using short tandem repeat profiling by ATCC, propagated, expanded and frozen immediately into numerous aliquots after arrival. The revived cells were utilized within 10 to 12 passages and not exceeding a period of 3 months and were cultured following the instructions of the company. Cells were regularly screened for mycoplasma contamination using MycoAlert Mycoplasma Detection Kits (Lonza). All supplements were obtained from Sigma (Saint Louis, MO, USA) except for antibiotic and antimycotic solutions, which were obtained from Gibco (Grand Island, NY, USA). Cells were maintained at 37 °C in humidified incubator with 5% CO_2_.

### Cellular lysate preparation and immunoblotting

This has been performed as previously described [13]. The anti-AUF1 antibody (07-260) was purchased from Millipore. Antibodies directed against Twist1 (10E4E6), IL-6, Snail (C15D3) were purchased from Abcam. ALDH1/2 (H-85), CD24 (C-20) and GAPDH (FL-335) were purchased from Santa Cruz Biotech (USA). E-cadherin (24E10), N-cadherin, OCT4 (C30A3), Sox2 (D6D9), STAT3 (124H6), p-STAT3 (Tyr705), KLF-4 (D1F2), cleaved PARP (Asp214), cleaved caspase-9 (Asp315), cyclinD1 (DCS6) and EpCAM (D1B3) were purchased from Cell Signaling Technology (USA). CD44 was purchased from Sigma-Aldrich. Anti-ZEB1 was purchased from Abnova and anti-vimentin from Abcam. ALDH1 was purchased from BD biosciences. All of these antibodies were used at 1:1000 dilution. Anti-IL-6 neutralizing antibody (6708-11) was purchased from Sigma-Aldrich.

### Cell proliferation, migration and invasion assays

These assays were performed in a label-free real-time setting using the xCELLigence RTCA technology (Roche, Germany) that measures impedance changes in a meshwork of interdigitated gold microelectrodes located at the well bottom (E-plate) or at the bottom side of a microporous membrane (CIM-plate 16). Cell migration and invasion were assessed as per the manufacturer’s instructions. In brief, 2 × 10^4^ cells in serum-free medium were added to the upper wells of the CIM-plate coated with a thin layer of Matrigel (BD Biosciences) basement membrane diluted 1:20 in serum-free medium (invasion) or non-coated (migration). Complete medium was used as a chemo-attractant in the lower chambers. Subsequently, the plates were incubated in the RTCA for 24 h and the impedance value of each well was automatically monitored by the xCELLigence system and expressed as Cell Index (CI) value, which represents cell status based on the measured electrical impedance change divided by a background value. Experiments were performed three times in triplicate.

For the proliferation assay, exponentially growing cells (2 × 10^4^) were seeded in E-plate with complete medium as per the manufacturer’s instruction. Cell proliferation was assessed for 48 h. All data were recorded and analyzed by the RTCA software. Cell Index was used to measure the change in the electrical impedance divided by background value, which represents cell status. Experiments were performed three times in triplicate.

### Spheroid formation

Cells were seeded in 96-well ultra-low attachment plate at density of 1000 viable cells/well. Cells were cultured in 171 medium supplemented with 1% ABM, 2% B-27, 20 ng/ml EGF, 500 ng/ml HC, 4% FBS and 5 μg/ml insulin. Cells were incubated for 10 days at 37 °C under 5% CO_2._ Mammospheres with diameter of ≥ 100 μm were counted using OPTIKA light microscope. Experiments were performed three times in triplicate.

### Conditioned media

Cells were cultured in medium without serum for 24 h, and then, media were collected and briefly centrifuged. The resulting supernatants were used either immediately or were frozen at − 80 °C until needed.

### Immunoadsorption of cytokine/antibody complex from SFCM

IL-6 present in the serum-free conditioned medium (SFCM) from TCF64-ORF cells was first inhibited by an IL-6 neutralizing antibody (2.5 μg/ml) (Sigma-Aldrich) for 3 h at 4 °C. IgG (R&D systems) was used as control. To deplete TCF64-ORF-SFCM from IL-6nAb as well as IL-6/IL-6nAb complex, A/G Sepharose (8 mg/ml) (BioVision) was added to SFCM and incubated overnight at 4 °C. The suspension was then centrifuged, and the supernatant was collected and filtered.

### Patients and archived clinical materials

Formalin-fixed paraffin-embedded tissues were obtained from the Pathology Department at KFSH&RC with institutional review board approval (RAC#2151051). The study cohort consisted of 344 patients (females) who were histologically diagnosed with unilateral locally advanced breast cancer (T2 ≥ 4 cm, T3 or T4, N0–N2, M0) of non-inflammatory nature. These patients were treated with neoadjuvant chemotherapy plus Trastuzumab when HER2 positive and definitive surgery and locoregional radiotherapy ± hormonal therapy, as previously described [14]. The enrolled patients were diagnosed between 2006 and 2013, with a median follow-up time of 52. 6 months. Written informed consent was not required, and a waiver was granted since samples were anonymized to the research team. Diagnosis of invasive breast cancer was done through true-cut needle biopsy. Immunohistochemistry of pre-treatment biopsy was used to determine estrogen (ER), progesterone receptors (PR) and HER2.

### Immunohistochemistry staining on FFPE tissues

Immunohistochemistry for AUF1 was performed on formalin-fixed paraffin-embedded tissues using anti-AUF1 antibody from Abcam (ab50692) overnight at a dilution of 1:500, and slides were stained using automated staining platform (Ventana). Envision + polymer (ready to use; Dako) was used as a secondary antibody. Color was developed with 3,3′-diaminobenzidine (DAB), and instant hematoxylin (Shandon) was used for counterstaining. The AUF1 level was evaluated and verified by two qualified pathologists, who scored both the proportion of positive cells and the intensity of AUF1 expression in both cancer cells and their stromal fibroblasts, and an immunoreactivity score was determined and used for statistical analysis.

### Flow cytometry

Cells (2.10^5^) were treated and then stained with CD44/CD24 as previously described [15]. Briefly, cells were washed and incubated with CD44 Pacific-Blue (from Invitrogen, USA) and CD24 PE (from BD Biosciences, USA) antibodies for surface staining (30 min at 4 °C). Data were acquired using the LSR II flow cytometer and the BD FACSDiva operating software. Positive staining was considered based on the negativity of an isotype control. A minimum of 10,000 events was recorded for all samples.

### Cytotoxicity assay

5000 cells/well were seeded in 96-well plates with appropriate culture media. After cells treatment, WST1 reagent (Sigma-Aldrich) was added to each well according to the manufacturer's instructions. These experiments were performed in triplicates and were repeated several times.

### Orthotopic tumor xenografts

Animal experiments were approved by the KFSH&RC institutional Animal Care and Use Committee (ACUC) and were conducted according to relevant national and international guidelines. Ten female nude mice were randomized into 2 groups, and breast cancer orthotopic xenografts were created by co-implantation of the MDA-MB-231 cells (2 × 10^6^) with TCF64-ORF or TCF64-CTRL cells (2 × 10^6^) under the nipple of each mouse. Tumor size was measured with a caliper using the following formula (Length X Width X Height).

### Statistical analysis

Statistical analysis was performed by the software package SAS version 9.4 (SAS Institute Inc., Cary, NC, USA). Continuous variables were compared by Student’s *t* test, and *P* values of 0.05 and less were considered as statistically significant. Kaplan–Meier method was used in survival tables and curves, and the different subgroups were compared by the log-rank test.

## Results

### Correlation of AUF1 expression in cancer cells and stromal fibroblasts with clinicopathological parameters

In the present study, we investigated the value of AUF1 expression level in both cancer cells and stromal fibroblasts as predictive biomarker for clinical outcome of LABC patients following neoadjuvant chemotherapy ± Trastuzumab. The clinicopathological features of the enrolled patients (*n* = 344) are listed in Additional file 1: Table S1. ER+/Her2+ patients (136) represented 39.5%, and ER+/Her2− (57) represented 16.5%, ER−/Her+ (78) represented 22.6%, while ER−/Her2− (73) represented 21.4%. Remarkably, most of the patients (73.84%) were less than 50 years old, and the same proportion had high tumor stage, while about 43% of the tumors were of grade 3. Great proportion of patients have developed recurrence (41%) and 74 succumbed to their disease (Additional file 1: Table S1). Notably, most tumors were of big size with T2 and T3 representing 76% (Additional file 1: Table S1).

**Fig. 1:**
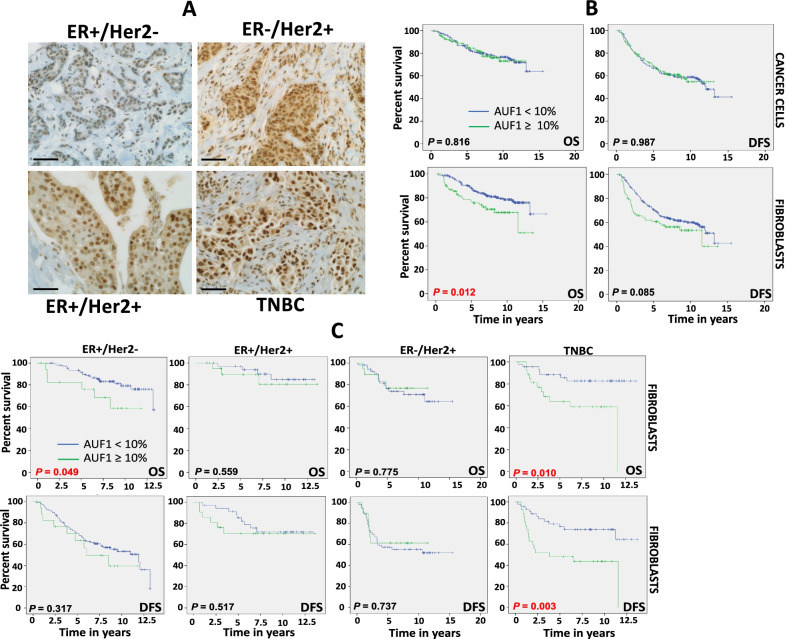
High AUF1 level in LABC tumors is associated with poor survival. **A** Tissue sections cut from formalin-fixed paraffin-embedded breast tumors (different subtypes) were immunostained with an anti-AUF1 antibody. The photographs were obtained using the light microscope Olympus BX53 (Envision 60x). Scale bar = 50 μm. **B**, **C** Kaplan–Meier analysis of overall survival (OS) and disease-free survival (DFS) relative to the level of AUF1 in fibroblasts

The AUF1 expression level was assessed in a total of 344 breast pre-treatment tumor tissues, in both cancer cells and their related stroma. AUF1 immunostaining was assessed in breast cancer tissues in both epithelium and stroma (Fig. 1A). The level AUF1 immunostaining was classified into 2 subgroups: low (< 10% AUF1 negative cells) and high (≥ 10%, AUF1-positive cells). Additional file 2: Table S2 shows that the AUF1 level was low or completely lost in 251/344 fibroblasts and 188/344 cancer cells, and high in 85/344 fibroblasts and 147/344 cancer cells. The analysis of AUF1 expression in the different subtypes showed correlation with ER/Her2. Indeed, high level of AUF1 in fibroblasts or cancer cells was significantly associated with lack of ER or Her2 (*P* = 0.0005 and *P* = 0.0296, respectively) (Additional file 2: Table S2). Table 1 shows no correlation between AUF1expression level in both CAFs and cancer cells and tumor size, tumor stage and tumor recurrence. However, high AUF1 expression in both types of cells was significantly associated with higher tumor grade (*P* = 0.021 (fibroblasts) and *P* = 0.0295 (cancer cells)) (Table 1).

**Table 1:** Correlations between AUF1 expression and clinicopathological characteristics in breast cancer patients

Parameter	Total (n = 338)	AUF1 in stromal fibroblasts		*P* value
	(%)	< 10	> 10	
Age				
< 50 years	247 (73.08)	182 (53.85)	65 (19.23)	0.5442
> 50 years	91 (26.92)	70 (20.71)	21 (6.21)	
Tumor size				
T2	78 (23.93)	57 (15.48)	21 (6.44)	
T3	116 (35.58)	90 (27.61)	26 (7.98)	0.5799
T4	132 (40.49)	95 (29.14)	37 (11.35)	
Stage				
II B	85 (26.07)	62 (19.02)	23 (7.06)	0.6988
III A	100 (30.67)	77 (23.62)	23 (7.06)	
III B	141 (43.25)	102 (31.29)	39 (11.96)	
Ki-67 index	335			
0	313 (93.43)	232 (69.25)	81 (24.18)	0.5924
< 15	3 (0.90)	3 (0.90)	0 (0)	
> 15	19 (5.76)	14 (4.18)	5 (1.49)	
Recurrence				
No	201 (59.47)	153 (45.27)	48 (14.20)	0.4242
Yes	137 (40.53)	99 (29.29)	38 (11.24)	
Grade				
Gl/well diff	5 (1.48)	5 (1.48)	0 (0)	
G2/moderately diff	169 (50.00)	134 (39.64)	35 (10.36)	0.0021
G3/poorly diff	144 (42.60)	94 (27.81)	50 (14.79)	
Gx/unknown	20 (5.92)	19 (5.62)	1 (0.30)	
Lymph nodes				
N0	36 (10.65)	28 (8.28)	8 (2.37)	0.5782
N1	182 (53.85)	139 (41.12)	43 (12.72)	
N2	87 (25.74)	60 (17.75)	27 (7.99)	
N3	33 (9.76)	25 (7.40)	8 (2.37)	
Progression				
No	223 (65.98)	170 (50.30)	53 (15.68)	0.3243
Yes	115 (34.02)	82 (24.26)	33 (9.76)	
Survival status				
Alive	265 (78.40)	203 (60.06)	62 (18.34)	0.0096
Dead	73 (21.60)	49 (14.50)	24 (7.10)	
Duration of clinical follow-up		7.8215	6.0165	

Parameter	Total (n = 337)	AUF1 in cancer cells		*P* value
	(%)	< 10%	> 10%	
Age				
< 50 years	246 (73.00)	145 (43.03)	101 (29.97)	0.0550
> 50 years	91 (27.00)	43 (12.76)	48 (14.24)	
Tumor size				
T2	78 (23.93)	40 (12.27)	38 (11.66)	
T3	116 (35.58)	67 (20.55)	49 (15.03)	0.6731
T4	132 (40.49)	73 (22.39)	132 (40.49)	
Recurrence				
No	200 (59.35)	108 (32.05)	92 (27.30)	0.4250
Yes	137 (40.65)	80 (23.74)	57 (16.91)	
Grade				
G1/well diff	5 (1.48)	4(1.19)	1 (0.30)	
G2/moderately diff	169 (50.15)	98 (29.08)	71 (21.07)	0.0295
G3/poorly diff	143 (42.43)	70 (20.77)	73 (21.66)	
Gx/unknown	20 (5.93)	16 (4.75)	4 (1.19)	
Stage				
II B	85 (26.15)	46 (14.15)	39 (12.00)	0.9205
III A	99 (30.46)	53 (16.31)	46 (14.15)	
III B	141 (43.38)	79 (24.31)	62 (19.08)	
Lymph nodes				
N0	36 (10.68)	14 (5.04)	19 (5.64)	
N1	182 (54.01)	107 (31.75)	75 (22.26)	0.2923
N2	86 (25.52)	43 (12.76)	43 (12.76)	
N3	33 (9.79)	21 (6.23)	12 (3.56)	
Progression				
No	222 (65.88)	116 (34.42)	106 (31.45)	0.0695
Yes	115 (34.12)	72 (21.36)	43 (12.76)	
Survival status				
Alive	264 (78.34)	145 (43.03)	119 (35.31)	0.5445
Dead	73 (21.66)	43 (12.76)	30 (8.90)	
Duration of clinical follow-up		8.0190	6.5356	

### AUF1 expression level predicts survival in LABC patients

Survival analysis shows clear association between the level of AUF1 in stromal cells and patient’s overall survival (OS) as well as disease-free survival (DFS) (Fig. 1B). Indeed, patients with tumors expressing high levels of AUF1 in fibroblasts had significantly poorer OS and DFS (Fig. 1B). However, patients with tumors expressing low AUF1 in CAFs showed better survival (*P* = 0.085 and *P* = 0.012, respectively) (Fig. 1B). No correlation was observed between AUF1 expression level in cancer cells and patient’s survival (Fig. 1B). Univariate Cox regression analysis also showed an increased risk for patients with high AUF1 levels in stromal fibroblasts (*P* = 0.0151) but not in cancer cells (Table 2). Next, multivariate Cox regression analysis was conducted and showed that the AUF1 expression level in stromal fibroblasts is a significant independent predictor of DFS and OS (*P* = 0.0249, *P* = 0.0315, respectively) (Table 3).

**Table 2:** Univariate Cox proportional regression analysis on 5-year overall and disease-free survival of 344 breast cancer patients

Parameter	Overall survival			Disease-free survival		
	Hazard ratio	95% Cl	*P* value	Hazard ratio	95% Cl	*P* value
AUF1 (fibroblasts)						
≥ 10%	1.000			1.000		
< 10%	0.544	0.333–0.889	0.0151	0.705	0.484–1.026	0.0677
AUF1 (cancer cells)						
≥ 10%	1.000			1.000		
< 10%	0.945	0.589–1.516	0.8152	0.969	0.688–1.366	0.8579
Tumor size						
T2	1.000			1.000		
T3	1.703	0.842–3.448	0.1387	1.353	0.826–2.216	0.2304
T4	2.296	1.168–4.516	0.016	1.95	1.223–3.109	0.005
Age						
≤ 50 years	1.000			1.000		
> 50 years	1.357	0.828–2.223	0.2257	0.946	0.645–1.388	0.7774
Lymph node						
N0	1.000			1.000		
N1	1.084	0.454–2.586	0.856	1.286	0.680–2.429	0.4392
N2	2.311	0.954–5.6	0.0636	2.187	1.13–4.230	0.0201
N3	1.39	0.466–4.145	0.5546	1.631	0.74–3.596	0.2251
Stage						
IIA	1.000			1.000		
IIIA	1.921	0.932–3.963	0.0771	2.257	1.332–3.822	0.0025
IIIB	2.612	1.336–5.106	0.005	2.67	1.624–4.389	0.0001
Grade						
G1	1.000			1.000		
GII	0.938	0.128–6.868	0.9495	0.869	0.212–3.554	0.845
GIII	1.336	0.183–9.762	0.7754	1.037	0.253–4.249	0.9597
ER/Her2 status						
ER(+ve)/Her2(+ve)	1.000			1.000		
ER(+ve)/Her2(−ve)	0.612	0.267–1.404	0.2458	0.522	0.297–0.917	0.0237
ER(-ve)/Her2(+ve)	1.52	0.855–2.699	0.1535	0.929	0.602–1.434	0.7393
ER(−ve)/Her2(−ve)	1.417	0.783–2.564	0.2494	1.025	0.667–1.575	0.9107

**Table 3:** Multivariate Cox regression analysis on 5-year overall and disease-free survival

Parameter	Overall survival			Disease-free survival		
	Hazard ratio	95% CI	*P* value	Hazard ratio	95% CI	*P* value
AUF1 (fibroblasts)	0.950	0.334–0.950	0.0315	0.634	0.634–0.426	0.0249
Tumor size						
T3	1.112	0.374–3.308	0.8480	0.403	0.174–0.931	0.0333
T4	0.756	0.160–3.578	0.7242	0.711	0.202–2.500	0.5952
Age (> 50 years)	1.059	0.609–1.840	0.8392	0.2040	0.489–1.165	0.755
Stage						
IIIA	1.934	0.534–6.997	0.3148	4.827	1.856–12.550	0.0012
IIIB	3.237	0.589–17.782	0.1765	3.789	0.970–14.796	0.0553
Grade						
G2	0.649	0.097–6.172	0.8100	0.641	0.150–2.738	0.5483
G3	0.775	0.050–5.410	0.5862	0.743	0.172–3.215	0.6907
Lymph node						
N1	0.855	0.283–2.589	0.7821	0.661	0.310–1.406	0.2823
N2	1.515	0.447–5.132	0.5049	0.890	0.387–2.048	0.7848
N3	0.923	0.206–4.135	0.9162	0.623	0.217–1.785	0.3782
HR_ Status						
ER(+ve)/Her2(−ve)	0.433	0.182–1.030	0.0584	0.502	0.502–0.275	0.0249
ER(−ve)/Her2(+ve)	1.353	0.714–2.564	0.3536	1.045	1.045–0.643	0.8594
ER(−ve)/Her2(−ve)	1.113	0.584–2.123	0.7448	1.013	1.013–2.231	0.9586
AUF1 (cancer cells)	1.042	0.629–1.726	0.8742	0.937	0.646–1.359	0.7305
Tumor size						
T3	1.148	0.380–3.468	0.8071	0.427	0.182–1.001	0.0504
T4	0.665	0.136–3.259	0.6150	0.736	0.202–2.681	0.6415
Age (> 50 years)	1.034	0.594–1.801	0.9051	0.734	0.475–1.134	0.1639
Stage						
IIIA	1.733	0.476–6.309	0.4041	4.327	1.652–11,335	0.0029
IIIB	3.585	0.629–20.426	0.1505	3.573	0.884–14.443	0.0740
Grade						
G2	0.716	0.091–5.651	0.7515	0.676	0.158–2.892	0.5977
G3	0.915	0.115–7.288	0.9330	0.818	0.189–3.546	0.7883
Lymph node						
N1	0.897	0.298–2.703	0.8467	0.701	0.330–1.488	0.3542
N2	1.664	0.498–5.552	0.4078	0.976	0.428–2.226	0.9539
N3	0.922	0.201–4.232	0.9167	0.668	0.232–1.925	0.4551
HR_ Status						
ER(+ve)/Her2(−ve)	0.466	0.196–1.104	0.0828	0.531	0.292–0.969	0.0390
ER(−ve)/Her2(+ve)	1.444	0.763–2.731	0.2590	1.089	0.670–1.768	0.7317
ER(−ve)/Her2(−ve)	1.162	0.608–2.220	0.6503	1.060	0.658–1.709	0.8104

Next, the AUF1 level was assessed in the 4 main breast cancer subtypes. Figure 1C shows a significant correlation between fibroblast AUF1-positivity and poor OS of ER+/Her2− and triple-negative breast cancer (TNBC) patients (*P* = 0.049 and *P* = 0.010, respectively). However, no correlation was observed with the other subtypes (Fig. 1C). AUF1 positivity in cancer cells showed association with poor OS and DFS of ER+/Her2+ patients (*P* = 0.085 and *P* = 0.183) (Additional file 3: Fig. S1). Figure 1C shows a very strong correlation between high fibroblast AUF1 level and poor DFS of the TNBC patients (P = 0.003). However, no correlation was observed with the other subtypes (Fig. 1C).

### Neoadjuvant therapy-dependent downregulation of AUF1 in tumors improves patient outcome

**Fig. 2:**
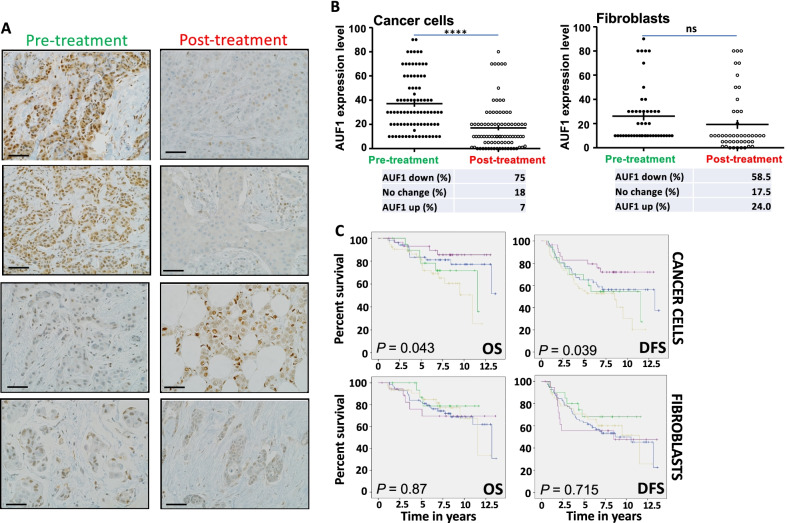
Neoadjuvant therapy-dependent downregulation of AUF1 in tumors improves patient survival. Tissue sections cut from paired FFPE obtained pre-treatment and post-treatment were immunostained with an anti-AUF1 antibody. **A** Selected photographs obtained by the light microscope Olympus BX53 (Envision 40x). Scale bar = 50 μm. **B** Graphs depicting the effect of neoadjuvant therapy on the expression level of AUF1. (****P < 0.0001). **C** Kaplan–Meier analysis of overall survival (OS) and disease-free survival (DFS). Purple: AUF1 level down to < 10%, blue: AUF1 level not affected, green: AUF1 level up to ≥ 10%, brown: AUF1 level up/down but did not reach 10%

Next, we sought to determine the effect of patient treatment on the expression of AUF1 in tumor and stromal cells. To this end, the expression of AUF1 was assessed on paired FFPE specimens collected pre- and post-treatment of each patient, for a total of 156 patients. The immunostaining results indicate downregulation, upregulation as well as no effect on the level of AUF1 in both cancer cells and their stromal adjacent fibroblasts (Fig. 2A). Indeed, in cancer cells, AUF1 was downregulated in 46% cases and increased in only 20% cases, while 34% cases showed no change in AUF1 immunoreactivity (Additional file 4: Fig. S2). In fibroblasts, the AUF1 level decreased in only 22% cases and increased in 33% cases, while 45% cases showed no change in AUF1 immunoreactivity (Additional file 4: Fig. S2). This shows that for several patients, the effect of the treatment on AUF1 expression varies between cancer cells and their associated fibroblasts. Next, we investigated the effect of the neoadjuvant treatment on the expression of AUF1 among the tumors with high AUF1 immunoreactivity (≥ 10%). Figure 2B shows that while AUF1 downregulation (to a level < 10%) was highly significant in cancer cells (75%), it was not significant in fibroblasts (58.5%). This indicates that neoadjuvant therapy can reduce the AUF1 level (< 10%) in malignant cells in a significant number of tumors. This prompted us to test whether this AUF1 downregulation to a low level (< 10%) could affect patient survival. Kaplan–Meier plots shown in Fig. 2C indicate association between AUF1 downregulation in cancer cells and patient’s disease-free survival (DFS) as well as overall survival (OS). Indeed, AUF1 downregulation to a low level (< 10%) improved the survival of the patients (Fig. 2C). However, no association was observed between patient’s survival and AUF1 downregulation in fibroblasts (Fig. 2C).

### Breast stromal fibroblasts that express high level of AUF1 promote carcinogenesis

**Fig. 3:**
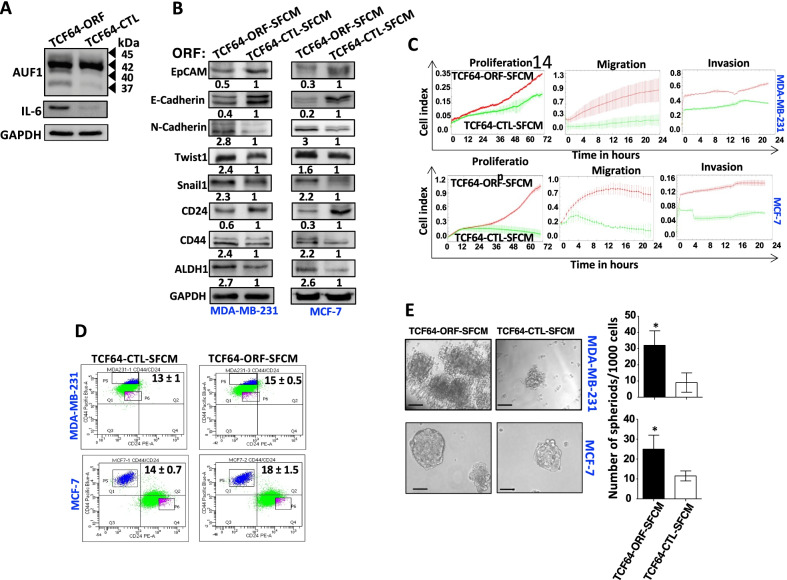
Ectopic expression of AUF1 in BSFs promotes carcinogenesis in a paracrine manner. **A** TCF-64 cells were transfected with vectors either empty (TCF64-CTL) or bearing p37^AUF1-ORF^ (TCF64-ORF). Whole cell lysates were prepared from these cells and were used for immunoblotting using antibodies against the indicated proteins. **B** TCF64-CTL and TCF64-ORF cells were cultured in SFM for 24 h, and the respective SFCM (TCF64-CTL-SFCM and TCF64-ORF-SFCM) were collected and were used to treat the indicated BC cells for 24 h. Whole cell lysates were prepared and were used for immunoblotting analysis. The numbers below the bands represent fold change relative to the corresponding control after correction against the internal control, GAPDH. **C** Exponentially growing cells were added in SFM to the upper wells of the CIM plates either separated by a Matrigel basement membrane matrix (invasion) or without (migration), or E-plate (proliferation), and then, these capacities were assessed for the indicated periods of time using the RTCA-DP xCELLigence System. Data are representative of different experiments performed in triplicate. **D** Cells were double-stained for CD24 and CD44, and the proportions of CD44^high^/CD24^low^ subpopulations were determined by flow cytometry and are indicated in the boxes as mean ± SD.; *n* = 3. **E** Cells (1000) were cultured in ultra-low attachment 96-well plates in the presence of specific stem cell medium. Left panel*,* representative images of mammospheres. Scale bar, 50 μm. Right panel, Graphs depicting the number of formed mammospheres. Experiments were performed in triplicate, and error bars represent means ± SD (*n* = 3) (*P < 0.05)

The AUF1-p37 isoform is the most active with the highest pro-carcinogenic effects in transgenic mice [16–19]. In order to elucidate the effect of AUF1 upregulation in breast stromal fibroblasts on breast carcinogenesis, we have first ectopically expressed AUF1 (p37) in normal breast fibroblasts (TCF64). TCF64 cells were infected with lentivirus-based vectors either empty (TCF64-CTL) or bearing the p37^AUF1-ORF^ (TCF64-ORF). Figure 3A shows upregulation of the 4 AUF1 isoforms in TCF64-ORF cells relative to controls. This could be mediated indirectly through the positive IL-6/STAT3 feedback loop [10]. Next, TCF64-ORF and TCF64-CTL were cultured in serum-free medium for 24 h, and then, serum-free conditioned media (SFCM) were collected, TCF64-ORF-SFCM and TCF64-CTL-SFCM, respectively. The obtained SFCM were applied on two BC cell lines MDA-MB-231 (triple-negative breast cancer cells) and MCF-7 (luminal breast cancer cells) for 24 h, and then, whole cell lysates were prepared for immunoblotting analysis. Figure 3B shows downregulation of the epithelial markers (EpCAM and E-cadherin) and upregulation of the mesenchymal markers (N-cadherin, Twist1 and Snail1) in BC cells that were exposed to TCF64-ORF-SFCM as compared to controls. This suggests that breast fibroblasts that express high level of AUF1 can enhance the EMT process in BC cells in a paracrine manner. This was confirmed by showing a clear increase in the proliferative, migratory and invasive capacities of these BC cells upon exposure to TCF64-ORF-SFCM (Fig. 3C). These findings prompted us to explore the possible promotion of stemness in these BC cells. Indeed, the exposure of both BC cell lines to TCF64-ORF-SFCM downregulated CD24 while upregulated CD44 and ALDH1 as compared to controls (Fig. 3B). Flow cytometric analysis showed TCF64-ORF-SFCM-dependent increase in the proportion of CD44^high^/CD24^low^ subpopulation of cells in MDA-MB-231 and MCF-7 cell lines, as compared to their respective controls (Fig. 3D). Furthermore, the number of the formed tumorspheres was higher in BC cells that were exposed to TCF64-ORF-SFCM than in their respective controls (Fig. 3E). These results indicate that BSFs that express high level of AUF1 have the capacity to promote EMT and stemness, two pro-metastatic processes, in breast cancer cells both ER^+^ and ER^−^.

### Breast stromal fibroblasts that express high level of AUF1 promote carcinogenesis in an IL-6-dependent paracrine manner

**Fig. 4:**
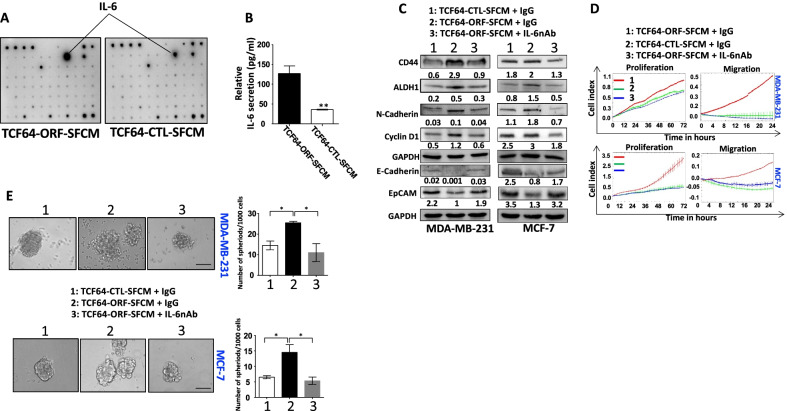
Ectopic expression of AUF1 in BSFs promotes carcinogenesis in an IL-6-dependent paracrine manner. **A** The indicated SFCM were applied onto human cytokine antibody array membranes (C7). **B** The indicated SFCM were used to assess the level of IL-6 by ELISA, and error bars represent mean ± SD (*n* = 3) (**P < 0.01). **C** Cells were treated as indicated for 24 h, and then, whole cell lysates were prepared and were used for immunoblotting analysis. The numbers below the bands represent fold change relative to the respective controls after correction against the internal control. **D** Cells were treated as indicated, and the migration and proliferation capacities were assessed for the indicated periods of time using the RTCA-DP xCELLigence System. Data are representative of different experiments performed in triplicate. **E** Cells (1000) were cultured in ultra-low attachment 96-well plates in the presence of specific stem cell medium. Left panel*,* representative images of mammospheres. Scale bar, 100 μm. Right panel, Graphs depicting the number of formed mammospheres. Experiments were performed in triplicate, and error bars represent means ± SD (*n* = 3) (*P < 0.05)

Next, we decided to determine the main factor responsible for the paracrine pro-carcinogenic promotion of BSFs that express high level of AUF1. To this end, TCF64-ORF-SFCM and TCF64-CTL-SFCM were applied on cytokine array, which showed higher secreted level of IL-6 in TCF64-ORF-SFCM than in TCF64-CTL-SFCM (Fig. 4A). This was confirmed by ELISA, which showed a three-fold increase in the level of the secreted IL-6 from AUF1-expressing cells relative to their controls (Fig. 4B). In order to confirm the role of BSF-secreted IL-6 in promoting EMT and stemness in BC cells, IL-6 was neutralized using a specific anti-IL-6 antibody in TCF64-ORF-SFCM, while IgG was used as negative control in both TCF64-ORF-SFCM and TCF64-CTL-SFCM. Interestingly, IL-6 inhibition in TCF64-ORF-SFCM upregulated the level of the epithelial markers E-cadherin and EpCAM, while it reduced the expression level of the mesenchymal marker N-cadherin and the stemness markers CD44 and ALDH1 as well as cyclin D1 in both MDA-MB-231 and MCF-7 BC cells (Fig. 4C). This IL-6 neutralization in TCF64-ORF-SFCM led to inhibition of cell proliferation, migration as well as self-renewal capacity of both MCF-7 and MDA-MB-231 as compared to the respective controls (Fig. 4D, E). This indicates that IL-6 mediates the pro-carcinogenic effects of BSFs that express high level of AUF1.

### Breast stromal fibroblasts that express high level of AUF1 promote breast tumor growth

**Fig. 5:**
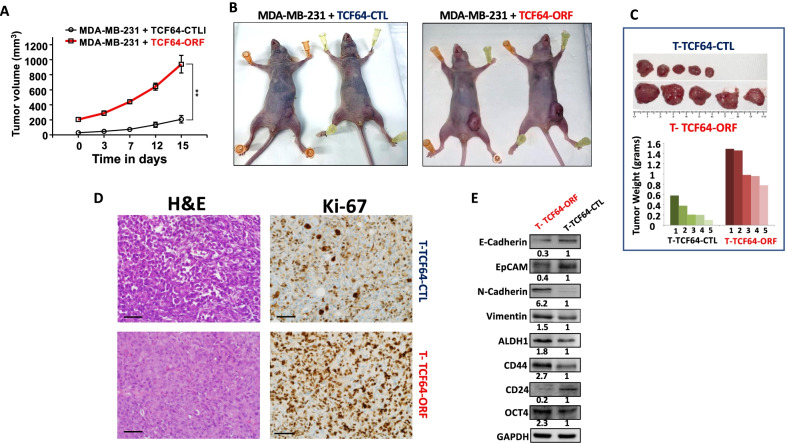
BSFs that express high level of AUF1 promote tumor growth in mice. **A** Orthotopic breast cancer xenografts were created by co-injecting MDA-MB-231 cells with TCF64-ORF or TCF64-CTL cells into the mammary fat pads of nude mice (*n* = 5). Graph depicting tumor volume over time and error bars indicate mean ± SEM (*n* = 5). **P ≤ 0.01. **B** Representative photographs showing the size of the formed tumors in 2 animals from each group. **C** Macroscopic view of xenograft tumors upon retrieval from mice (top panel). Histogram depicting the weight of the tumors in each group (bottom panel). **D** Representative photographs of histologic sections subjected to hematoxylin and eosin staining and to anti‐Ki-67 antibody. Scale bar = 50 μm. **E** Protein extracts were prepared from the excised tumors and were used for immunoblotting analysis using specific antibodies against the indicated proteins. The numbers below the bands indicate fold changes relative to the control (T-TCF64-CTL) after normalization against GAPDH

To further show the paracrine pro-carcinogenic effects of AUF1-expressing fibroblasts, we decided to test this effect in vivo using orthotopic tumor xenografts. To this end, TCF64-ORF and TCF64-CTL cells (2.10^6^) were co-injected with MDA-MB-231 cells (2.10^6^) into the fat pad of nude mice. Interestingly, palpable tumors containing TCF64-ORF-SFCM (T-TCF64-ORF) cells were detected 2 weeks post-injection, while those containing the control cells (T-TCF64-CTL) became visible 3 weeks post-injection (Fig. 5A). The graph shows also that the T-TCF64-ORF tumors grew faster than their corresponding controls (Fig. 5A). The pictures of two obtained tumors from each group are depicted in Fig. 5B, which shows that the tumors bearing TCF64-ORF-SFCM cells are much bigger than the control tumors. Next, tumors were excised and weighed and whole cell lysates were prepared. Interestingly, all the T-TCF64-ORF tumors are bigger and heavier than the T-TCF64-CTL tumors (Fig. 5C). Hematoxylin and eosin staining on paraffin-embedded sections showed more necrosis in T-TCF64-CTL compared to T-TCF64-ORF. Moreover, Ki-67 level was higher in T-TCF64-ORF than in the respective controls (Fig. 5D). This indicates that orthotopic breast tumors containing TCF64-ORF grew faster owing to their higher proliferative capacity than their corresponding control tumors. Figure 5E shows that the presence of TCF64-ORF cells enhanced the expression of the mesenchymal markers N-cadherin and vimentin and reduced the expression of the epithelial markers EpCAM and E-cadherin. Furthermore, T-TCF64-ORF tumors expressed lower level of CD24 and higher levels of the stemness makers CD44, ALDH1 and OCT4 (Fig. 5E). This indicates that the TCF64-ORF cells promoted the mesenchymal and the stemness features in the humanized breast cancer tumors in mice.

### Breast stromal fibroblasts that express high level of AUF1 promote chemoresistance in breast cancer cells

**Fig. 6:**
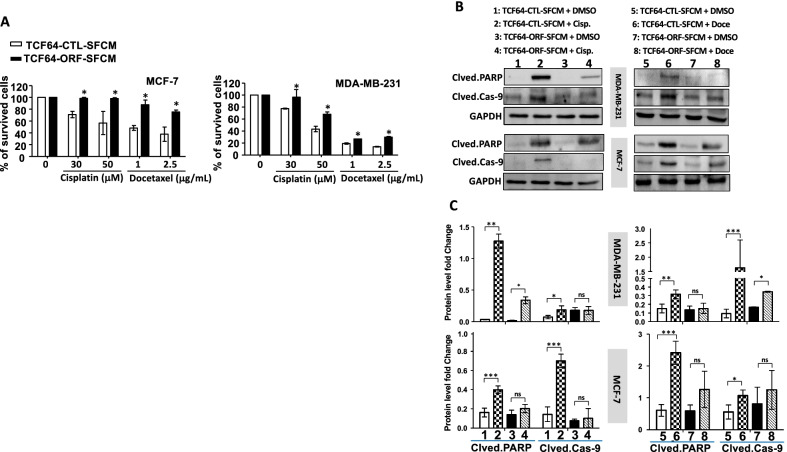
BSFs that express high level of AUF1 confer chemoresistance to BC cells in a paracrine fashion. **A** MDA‐MB‐231 and MCF-7 cells (5 × 10^3^ cells per well) were seeded in 96‐well plates and were incubated with TCF64-ORF-SFCM or TCF64-CTL-SFCM for 24 h and then were either sham-treated or challenged with different concentrations of cisplatin (30 and 50 μM) or docetaxel (1 and 2.5 μg/ml) for 72 h. Cell cytotoxicity was assessed using the WST1 assay and recorded as percent of survived cells relative to the untreated cells. Error bars indicate mean ± SEM (*n* = 3). *P ≤ 0.05. **B** Cells were incubated with TCF64-ORF-SFCM or TCF64-CTL-SFCM for 24 h and then were either sham-treated (DMSO) or challenged with cisplatin (30 μM) or docetaxel (2.5 μg/ml) for 72 h. Whole cell lysates were prepared and were used for immunoblotting analysis using antibodies against the indicated proteins. The numbers below the bands indicate fold changes relative to the respective control after normalization against GAPDH. **C** Quantifications of the immunoblotting results depicted in Fig. 5B. Error bars indicate mean ± SEM (*n* = 3). *P ≤ 0.05, **P ≤ 0.01, ***P ≤ 0.001, ****P ≤ 0.0001, ns: not significant

Next, we sought to assess the effect of BSFs that express high level of AUF1 on the response of breast cancer cells to chemotherapeutic drugs. To this end, we incubated MDA-MB-231 and MCF-7 cells with SFCM from TCF64-ORF or its corresponding control TCF64-CTL for 24 h, and then, cells were either sham-treated or challenged with cisplatin (30 and 50 μM) or docetaxel (1 and 2.5 μg/ml) for 72 h. The WST1 assay was performed to evaluate the cytotoxicity of both drugs. Figure 6A shows that exposing BC cells to TCF64-ORF-SFCM resulted in a significant increase in their resistance to both cisplatin and docetaxel as compared to controls.

To confirm these findings at the molecular level, immunoblotting analysis was performed to evaluate the level of the important pro-apoptotic proteins PARP and caspase-9 in pre-treated BC cells. The obtained results indicate that TCF64-ORF-SCFM reduced the cisplatin- and docetaxel-dependent increase in the level of cleaved PARP and cleaved caspase-9 as compared to controls for both cell lines (Fig. 6B, C). These results reveal that BSFs that express high level of AUF1 protect breast cancer cells from the cytotoxic effects of cisplatin and docetaxel in a paracrine manner.

## Discussion

LABC, the most aggressive BC subtype, remains a challenging clinical problem in most developing countries with high disease relapse and poor survival rates [5]. In order to improve the treatment of these patients, it is crucial to identify biomarkers with efficient predictive value. Thereby, we sought in the present study to evaluate the prognostic/predictive power of the expression level of AUF1 in patients suffering LABC. We have first shown significant association between high AUF1 level in both CAFs and cancer cells and high tumor grade (*P* = 0.0021 and *P* = 0.0295, respectively). Furthermore, high AUF1 level in fibroblasts was associated with poor DFS and OS, which was confirmed by univariate Cox regression analysis. Moreover, multivariate Cox regression analysis indicated that the AUF1 level in stromal fibroblasts is a significant independent predictor of DFS and OS (*P* = 0.0249, *P* = 0.0315, respectively). On the other hand, AUF1 level in cancer cells was not associated with survival of LABC patients.

When AUF1 level was assessed in the four well-defined breast cancer subtypes, we have found that AUF1-positive stromal fibroblasts were significantly correlated with poor survival of both ER+/Her2− and ER−/Her2− patients (Fig. 1C). Similarly, high expression of AUF1 in malignant cells tended to be associated with shorter survival of ER+/Her2+ patients. These findings indicate that the expression level of AUF1 in breast CAFs has a powerful prognostic value for TNBC as well as ER+/Her2− patients. In fact, several lines of evidence point to the key role of CAFs in modulating the response of breast cancer patients to various types of therapies [20].

Additionally, we have observed significant neoadjuvant therapy-dependent change in the expression of AUF1 in malignant cells, with AUF1 downregulation in 46% of cases. Importantly, AUF1 downregulation to a level lower than 10% (cutoff) in tumors was significantly associated with better OS and DFS (*P* = 0.043 and *P* = 0.039, respectively). However, this decrease was not significant in CAFs, and therefore, it did not improve survival. This shows the importance of developing specific anti-AUF1 inhibitors for more efficient precision medicine.

It has been recently shown that AUF1 is highly expressed in colorectal cancer tissues and cell lines and this was associated with a poor prognosis [21]. This shows that the importance of AUF1 as prognostic biomarker may not be limited to BC but could be also highly informative for other types of cancer.

We have previously shown that AUF1 is highly expressed in cancer-associated fibroblasts compared to their paired counterparts, and also, AUF1 plays a major role in the activation of BSFs [10, 22]. Thereby, in order to provide a molecular explanation as to the association between high AUF1 expression in CAFs and the unfavorable prognosis, we investigated the paracrine effects of BSFs that ectopically express AUF1 on breast cancer cells. We have shown that AUF1 upregulation in BSFs promotes EMT and stemness in breast cancer cells in an IL-6-dependent manner. More importantly, AUF1-expressing BSFs enhanced the resistance of BC cells to the cytotoxic and pro-apoptotic effects of platinum as well as docetaxel chemotherapeutic agents. This indicates that AUF1 upregulation in BSFs promotes carcinogenesis and also reduces the response of BC cells to various chemotherapeutic agents, which explains the poor outcome of patients with tumors expressing high level of AUF1 in their stromal fibroblasts (Fig. 1). This suggests that breast fibroblast AUF1 could be considered as a novel prognostic biomarker, which could also be specifically targeted for precision therapy of BC patients.

The prognostic power of breast CAFs has been previously shown based on the expression level of different genes such as CAV-1, α-SMA, FAP-α, podoplanin and others [23–25]. Furthermore, we have recently shown that ATR-negative CAFs predict a poor OS as well as DFS for breast cancer patients [26].

## Conclusions

Together, these findings indicate that breast CAFs have important predictive/prognostic power that should be taken into consideration for the stratification and precise treatment of these patients, especially the hard-to-treat form of the disease (TNBC). This is due to the fact that these genetically stable and relatively abundant non-cancerous stromal cells have the ability to promote tumor growth, progression as well as the response to various anticancer agents [9]. This also indicates that personalized CAFs-targeted therapies may enable more efficient therapeutic responses.

## Supplementary Information

**Additional file 1: Table S1.** Clinicopathological characteristics of breast cancer patients by ER/Her2 subtypes (percentage).

**Additional file 2: Table S2.** Expression of AUF1 in cancer cells and stromal fibroblasts by breast cancer ER/Her2 subtypes.

**Additional file 3: Fig. S1.** AUF1 level in cancer cells does not affect patients’ survival. Kaplan–Meier analysis of overall survival (OS) and disease-free survival (DFS) relative to the level of AUF1 in cancer cells.

**Additional file 4: Fig. S2.** Neoadjuvant therapy modulates the expression of AUF1 in cancer and fibroblast cells. Tissue sections cut from formalin-fixed paraffin embedded breast tumors obtained pre-treatment and post-treatment, were immunostained with an anti-AUF1 antibody. Graphs depicting the effect of neoadjuvant therapy on the expression level of AUF1.

## Data Availability

The data generated, used and analyzed in the current study are available from the corresponding author in response to reasonable request.

## Abbreviations

CAFs: Cancer-associated fibroblasts
CSCs: Cancer stem cells
EMT: Epithelial-to-mesenchymal transition
ORF: Open reading frame
NBFs: Normal breast fibroblasts
TCFs: Tumor counterpart fibroblasts
TME: Tumor microenvironment
