# Supplementary material for: High AUF1 level in stromal fibroblasts promotes carcinogenesis and chemoresistance and predicts unfavorable prognosis among locally advanced breast cancer patients
Source: Breast Cancer Res. 2022 Jul 11;24:46. doi: 10.1186/s13058-022-01543-x (PMC9275022; doi:10.1186/s13058-022-01543-x)
Supplement: Supplementary file 1 — Additional file 1: Table S1. Clinicopathological characteristics of breast cancer patients by ER/Her2 subtypes (percentage). [file 13058_2022_1543_MOESM1_ESM.docx]

Supplementary Table S1. Clinicopathological characteristics of breast cancer patients by ER/Her2 subtypes (percentage)

| **Parameter** | **Total (n= 344) (%)** | **ER (+ve)/Her2 (+ve)** | **ER (+ve)/Her2 (-ve)** | **ER (-ve)/Her2 (+ve)** | **ER (-ve)/Her2 (-ve)** | ***P* value** |
| --- | --- | --- | --- | --- | --- | --- |
| **Age (years** |  |  |  |  |  |  |
| ≤50 | 254 (73.84) | 98 (28.49) | 40 (11.63) | 63 (18.31) | 53 (15.41) | 0.4547 |
| ˃50 | 90 (26.16) | 38 (11.05) | 17 (4.94) | 15 (4.36) | 20 (5.81) |  |
| **Tumor size** |  |  |  |  |  |  |
| T2 | 79 (23.80) | 35 (10.54) | 3 (0.9) | 23 (6.93) | 18 (5.42) |  |
| T3 | 117 (35.24) | 51 (15.36) | 26 (7.83) | 24 (7.23) | 16 (4.82) | 0.0021 |
| T4 | 136 (40.96) | 46 (13.86) | 25 (7.53) | 27 (8.13) | 38 (11.45) |  |
| **Recurrence** |  |  |  |  |  |  |
| No | 203 (59.01) | 72 (20.93) | 42 (12.21) | 47 (13.66) | 42 (12.21) | 0.0640 |
| Yes | 141 (40.99) | 64 (18.60) | 15 (4.36) | 31 (9.01) | 31 (9.01) |  |
| **Grade** |  |  |  |  |  |  |
| G1/well diff. | 5 (1.45) | 5 (1.46) | 0 (0) | 0 (0) | 0 (0) |  |
| G2/moderately diff. | 173 (50) | 82 (23.84) | 32 (9.30) | 23 (6.69) | 36 (10.47) | <.0001 |
| G3/poorly diff.  Gx/unknown | 148 (42.77)  20 (5.78) | 33 (9.59)  16 (4.65) | 24 (6.98)  1 (0.29) | 54 (15.70)  1 (0.29) | 36 (10.47)  1 (0.29) |  |
| **Stage**  II B  III A  III B | 87 (26.05)  102 (30.54)  145 (43.41) | 34 (10.24)  44 (13.25)  51 (15.36) | 8 (2.41)  21 (6.33)  27 (8.13) | 27 (8.13)  21 (6.33)  29 (8.73) | 16 (4.82)  16 (4.82)  38 (11.45) | 0.0627 |
| **Lymph nodes**  N0  N1  N2  N3 | 38 (10.98)  186 (53.76)  89 (25.72)  33 (9.54) | 18 (5.23)  78 (22.67)  25 (7.27)  15 (4.36) | 4 (1.16)  27 (7.85)  21 (6.10)  5 (1.45) | 12 (3.49)  44 (12.79)  18 (5.23)  4 (1.16) | 3 (0.87)  36 (10.47)  25 (7.27)  9 (2.62) | 0.0409 |
| **Progression** |  |  |  |  |  |  |
| No | 227 (65.99) | 81 (23.55) | 46 (13.37) | 53 (15.41) | 47 (13.66) | 0.0417 |
| Yes | 117 (34.01) | 55 (15.99) | 11 (3.20) | 25 (7.27) | 26 (7.56) |  |
| **Survival Status** |  |  |  |  |  |  |
| Alive | 270 (78.49) | 108 (31.40) | 50 (14.53) | 57 (16.57) | 55 (15.99) | 0.1930 |
| Dead | 74 (21.51) | 28 (8.14) | 7 (2.03) | 21 (6.10) | 18 (5.23) |  |
| **Duration of clinical follow-up (Years, mean±SD)** |  | 7.92 | 7.75 | 6.67 | 6.87 |  |
