# Supplementary material for: High AUF1 level in stromal fibroblasts promotes carcinogenesis and chemoresistance and predicts unfavorable prognosis among locally advanced breast cancer patients
Source: Breast Cancer Res. 2022 Jul 11;24:46. doi: 10.1186/s13058-022-01543-x (PMC9275022; doi:10.1186/s13058-022-01543-x)
Supplement: Supplementary file 2 — Additional file 2: Table S2. Expression of AUF1 in cancer cells and stromal fibroblasts by breast cancer ER/Her2 subtypes. [file 13058_2022_1543_MOESM2_ESM.docx]

**Supplementary Table S2**. Expression of AUF1 in cancer cells and stromal fibroblasts by breast cancer ER/Her2 subtypes

| **Epithelial cells** | **Total (%)** | **ER (+ve)/Her2 (+ve)** | **ER (+ve)/Her2 (-ve)** | **ER (-ve)/Her2 (+ve)** | **ER (-ve)/Her2 (-ve)** | ***P* value** |
| --- | --- | --- | --- | --- | --- | --- |
| **AUF1 (Fibroblasts)** |  |  |  |  |  |  |
| <10 | 251 (74.70) | 113 (33.63) | 36 (10.71) | 48 (14.29) | 54 (16.07) | 0.0005 |
| ≥10 | 85 (25.30) | 18 (5.36) | 21 (6.25) | 27 (8.04) | 19 (5.65) |  |
| **AUF1 (Tumor cells)** |  |  |  |  |  |  |
| <10 | 188 (56.12) | 76 (22.69) | 28 (8.36) | 34 (10.15) | 50 (14.93) | 0.0296 |
| ≥10 | 147 (43.88) | 55 (16.42) | 29 (8.66) | 40 (11.94) | 23 (6.87) |  |
