# Supplementary figures and images for: High AUF1 level in stromal fibroblasts promotes carcinogenesis and chemoresistance and predicts unfavorable prognosis among locally advanced breast cancer patients
Source: Breast Cancer Res. 2022 Jul 11;24:46. doi: 10.1186/s13058-022-01543-x (PMC9275022; doi:10.1186/s13058-022-01543-x)

Supplementary Figure S1

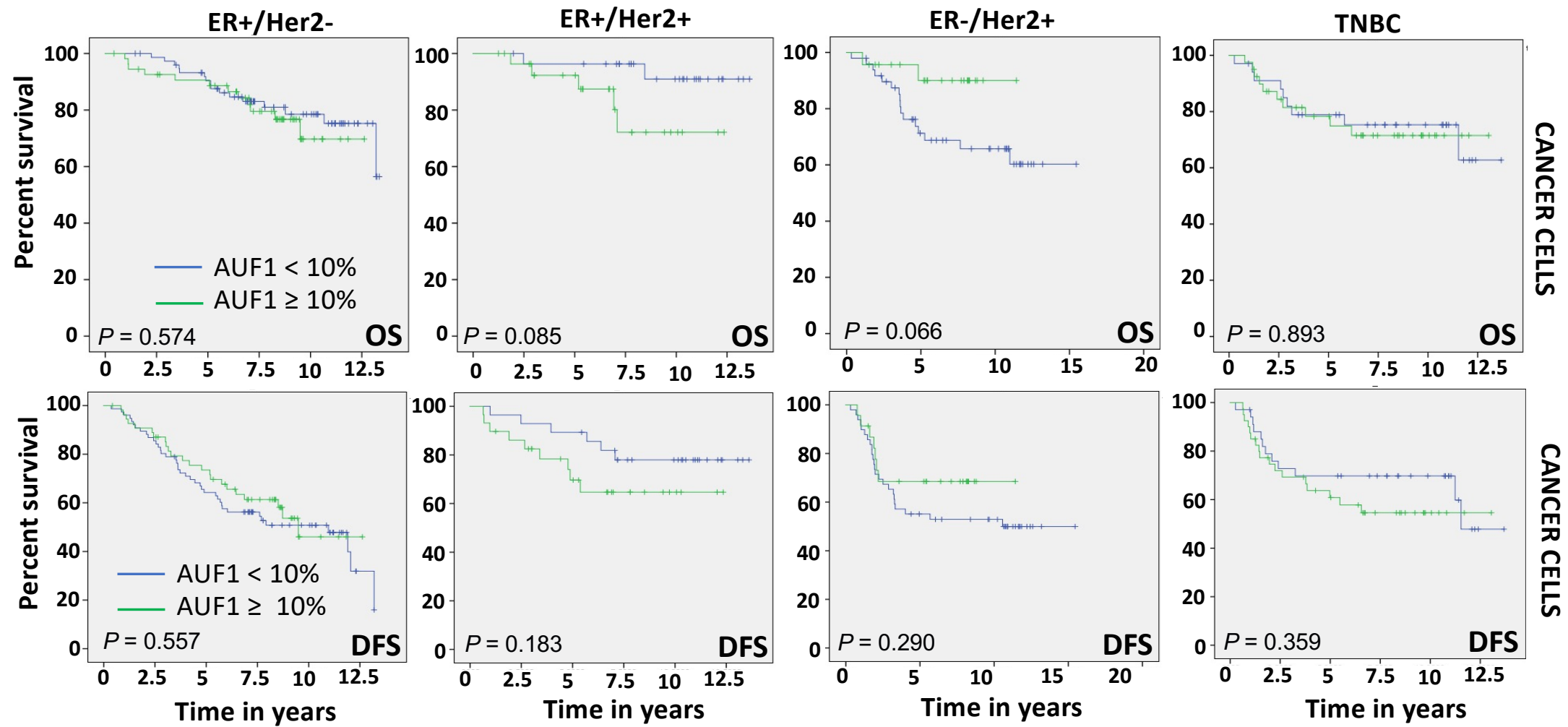

Supplement: Supplementary file 3 — Additional file 3: Fig. S1. AUF1 level in cancer cells does not affect patients’ survival. Kaplan–Meier analysis of overall survival (OS) and disease-free survival (DFS) relative to the level of AUF1 in cancer cells. [file 13058_2022_1543_MOESM3_ESM.pdf]

Supplementary Figure S2

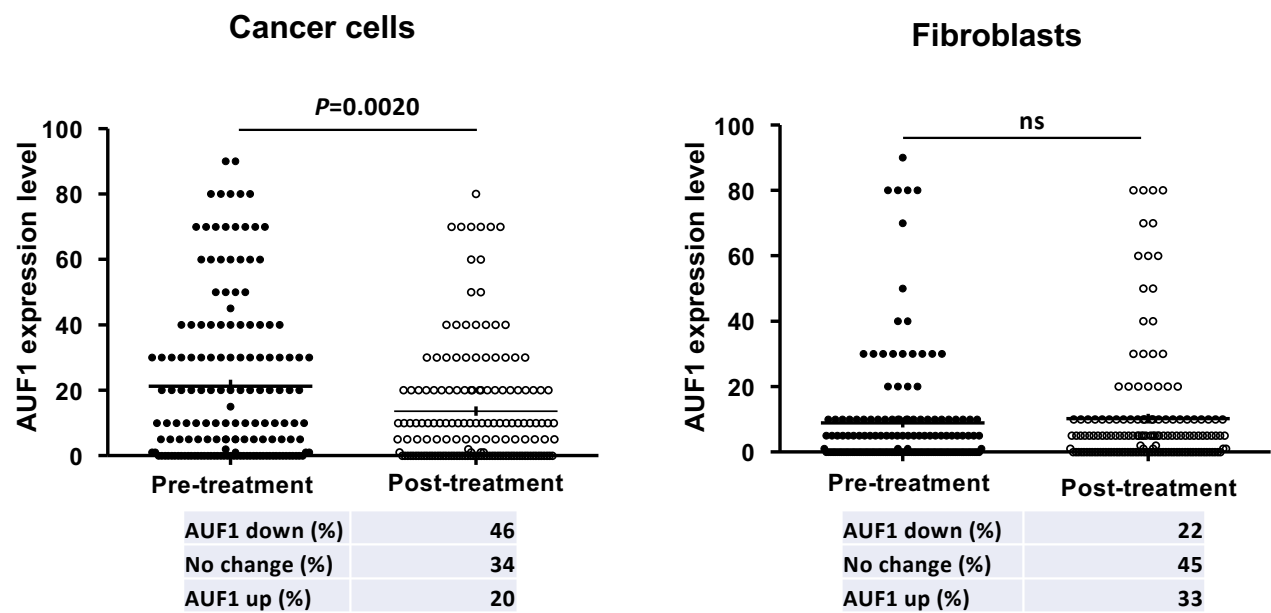

Supplement: Supplementary file 4 — Additional file 4: Fig. S2. Neoadjuvant therapy modulates the expression of AUF1 in cancer and fibroblast cells. Tissue sections cut from formalin-fixed paraffin embedded breast tumors obtained pre-treatment and post-treatment, were immunostained with an anti-AUF1 antibody. Graphs depicting the effect of neoadjuvant therapy on the expression level of AUF1. [file 13058_2022_1543_MOESM4_ESM.pdf]
